# Quick Response Code Verification Using Anti-Counterfeiting Pattern and Multi-Feature Fusion Network

**DOI:** 10.3390/s26103067

**Published:** 2026-05-12

**Authors:** Ke Sun, Zhongyuan Guo, Hong Zheng

**Affiliations:** 1 Jiatongda Technology (Hubei) Co., Ltd., Wuhan 430070, China; sk5288@163.com; 2 College of Computer Science and Engineering, Chongqing University of Science & Technology, Chongqing 401331, China; 3 Electronic Information School, Wuhan University, Wuhan 430072, China; zh@whu.edu.cn

**Keywords:** quick response code, anti-counterfeiting pattern, verification, multi-feature fusion

## Abstract

Quick response codes are widely used as anti-counterfeiting labels in the field of product packaging, but they are easily illegally copied. Thus, this paper introduces a quick response code verification method that combines an anti-counterfeiting pattern with a deep feature fusion network. Firstly, a specialized anti-counterfeiting quick response code is designed, composed of a standard quick response code and an anti-counterfeiting pattern, which is essentially a fine-grained random texture distribution sensitive to copying. Next, the anti-counterfeiting patterns are overlapped and divided into blocks during the data processing, which effectively expands the data volume and avoids the interference of pattern content on the authenticity identification. Then, a convolutional self-learning preprocessing layer is employed to initially learn the feature information that represents the difference between authenticity and forgery. Finally, a multi-feature fusion convolutional neural network is proposed to identity the authenticity of anti-counterfeiting patterns. The proposed network comprises two branches, facilitating multi-scale feature extraction and fusion. The effectiveness of the proposed approach is evaluated on a self-constructed quick response code dataset, and the experimental results demonstrate that the proposed approach outperforms traditional knowledge engineering methods and similar deep learning methods.

## 1. Introduction

Rapid economic development has greatly enriched people’s lives, but it has also brought many ensuing problems. Counterfeit and substandard goods are so diverse that they account for 3.3% of total global trade, and their value even reaches hundreds of billions of USD [1,2,3]. The proliferation of counterfeit goods has greatly endangered the development and survival of enterprises, disrupted the operation of the market economy, and caused huge economic losses to consumers [4,5]. Anti-counterfeiting technology [6,7,8,9] is an important means to deal with these counterfeit products. Consumers can quickly identify the authenticity of products through anti-counterfeiting labels, thus maintaining the brand image of the goods. Traditional anti-counterfeiting technologies include laser holography [10,11], physical labels [12,13], and special inks [14,15]. These technologies require special materials or unique formulas, resulting in high costs. Once the materials or formulas are leaked, the anti-counterfeiting properties are easily lost. Currently, quick response (QR) codes [16,17,18,19] are widely used in the field of product anti-counterfeiting due to their advantages, such as large information storage capacity, low printing costs, ease of production and durability. Consumers can scan the QR codes to verify the authenticity of goods and trace the supply chain. However, QR codes are easy to illegally copy. To solve this problem, researchers at home and abroad have conducted a series of studies:

Nguyen [20] proposed a watermarking anti-counterfeiting technology, which embeds randomly distributed fine textures in the QR code as a security layer. Any degradation process caused by forgery will change the distribution of micro-textures, and then a statistical detector is used under the framework of hypothesis testing for authenticity identification of printed QR codes. Picard [21] developed a secure QR code with a Copy Detection Pattern (CDP). CDP is essentially a random digital image and is sensitive to illegal copying. According to the above characteristics, effective anti-counterfeiting can be achieved by using an image registration algorithm. However, the above method has high requirements for pattern registration conditions and often leads to misjudgment in the case of poor registration. Yan [22] designed an IoT anti-counterfeiting system by combining visual features with QR codes. The visual features consider not only the natural texture features, but also the printed microscopic features. In the anti-counterfeiting verification process, the anti-counterfeiting verification results are obtained by comparing the similarity between the sample to be tested and the samples in the sample library through the feature extraction and comparison algorithm, which requires uploading all the authentic samples. Berenguel [23] used the texture analysis method to extract corresponding features from the anti-counterfeiting printed patterns to be tested, and designed an end-to-end mobile server to provide verification services for the most common users. However, the proposed method has high requirements for image clarity and requires special equipment for data collection, and it is difficult to achieve high-precision recognition performance on mobile phones. Chen [24] proposed to combine spectral and spatial features in a cascaded manner to design a QR code anti-replication scheme and achieved good identification results, but the feature extraction process is relatively complicated. Zheng [25] proposed an effective anti-counterfeiting pattern authentication method called the circumferential local ternary pattern, which employs random features extracted from inkjet prints to create local texture descriptors that are effective against noise and light interference, thus establishing a robust anti-counterfeiting authentication scheme.

In recent years, due to the substantial increase in training resources and the improvement in computing power, deep learning [26,27] has been applied to image forensics [28,29,30], and breakthroughs have been made in tasks such as print source identification [31,32], camera source identification [33,34,35,36], and tamper detection [37,38]. Convolutional neural network (CNN) [39,40] is one of the representative methods of deep learning. It uses data-driven methods for feature extraction and obtains more efficient deep feature representation. Guo [41] designed a convolutional neural network with a bottleneck residual module to identify the printer source of a QR code and then distinguish the authenticity, but it needs to collect data through special equipment and is only effective in closed sets. Zheng [42] proposed a two-stage anti-counterfeiting pattern authentication method fusing deep feature extraction and Support Vector Machine, which utilizes the feature extraction module in U-Net for feature extraction, and then inputs the features into a single classifier; in the second stage, a boundary-optimized OCSVM classification method is used, which requires only positive samples for effective recognition.

**Figure 1:**
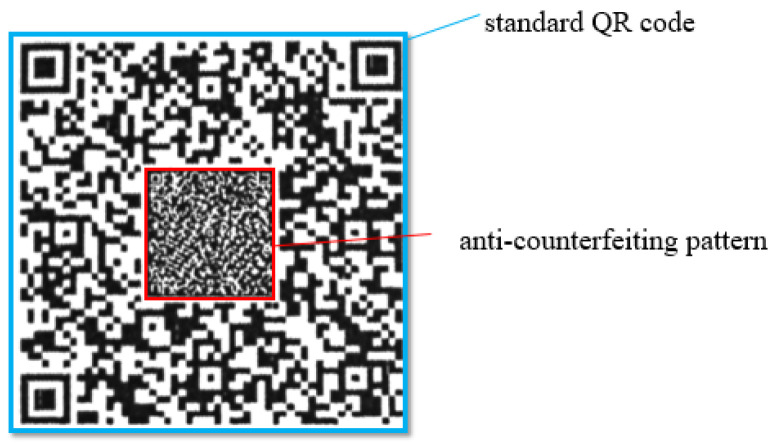
Composition of the anti-counterfeiting QR code.

**Figure 2:**
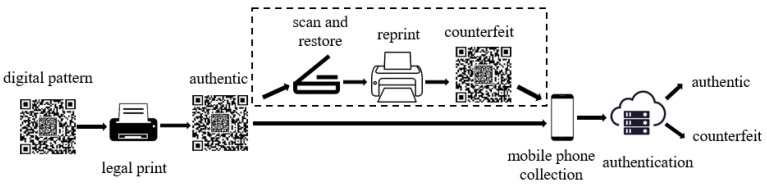
Production and counterfeiting process for an anti-counterfeiting QR code.

This paper studies an anti-counterfeiting QR code with random and subtle textures. which mainly consist of a standard QR code and an Anti-Counterfeiting Pattern (ACP), as shown in Figure 1. It possesses rich texture details and effectively improves anti-copying capabilities while maintaining the advantages of QR codes, such as universality, low cost, and ease of production. Figure 2 illustrates the production and counterfeiting process of the anti-counterfeiting QR code. The merchant first uses a digital generation system to generate the digital pattern of the anti-counterfeiting QR code, and then legally prints the genuine anti-counterfeiting QR code using an official authorized printer. Counterfeiters scan the digital image based on the authentic anti-counterfeiting QR code, recover it, and reprint it to obtain a counterfeit.

**Figure 3:**
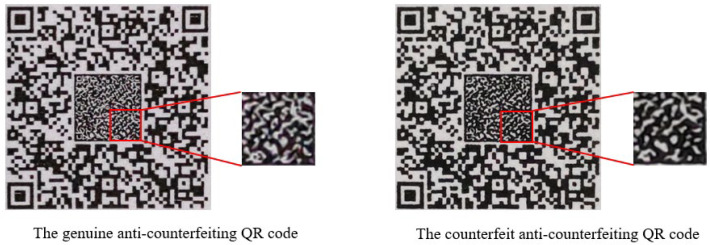
Differences in anti-counterfeiting patterns between real anti-counterfeiting QR codes and fake anti-counterfeiting QR codes.

During the production process of the anti-counterfeiting QR code, the toner will be randomly scattered to different degrees, resulting in irreproducible distortion, and the ACP is a random fine texture distribution that will further amplify the distortion changes. Figure 3 shows the difference between a genuine and counterfeit anti-counterfeiting QR code. Since the forgery process has to go through the process of scanning and reprinting, compared with the ACP of the genuine anti-counterfeiting QR code, the counterfeit ACP has rougher lines and more serious deformation, which can be used as the basis for authenticity identification.

Then, we proposed a CNN named Double-branch Multi-feature Fusion Network (DMFNet) for anti-counterfeiting QR code authentication. The preprocessing layer in the dual-branch architecture is used to fully amplify the difference between genuine and counterfeit ACPs, while the multi-scale feature extraction is completed by setting convolution kernels in the preprocessing layers, and the extracted multi-scale features are fused using the dual-branch architecture. The anti-counterfeiting QR codes are collected stably and clearly through smartphone devices, and the corresponding dataset is made for experiments. The results verify the effectiveness of the proposed method.

To sum up, the main contributions are summarized as follows:

(1) An anti-counterfeiting QR code is designed, primarily composed of a QR code and an anti-counterfeiting pattern, with the latter essentially consisting of a randomized intricate texture distribution sensitive to replication.
(2) A dual-branch multi-scale feature fusion network is proposed, and its feature extraction and fusion mechanism can effectively capture the forgery traces of the anti-counterfeiting pattern so as to verify the anti-counterfeiting QR code.
(3) The proposed scheme is tested on the self-built anti-counterfeiting QR code dataset, and the superiority of the proposed method is verified by ablation experiments and comparison methods.

## 2. Materials and Methods

### 2.1. The Overall Identification Process

**Figure 4:**
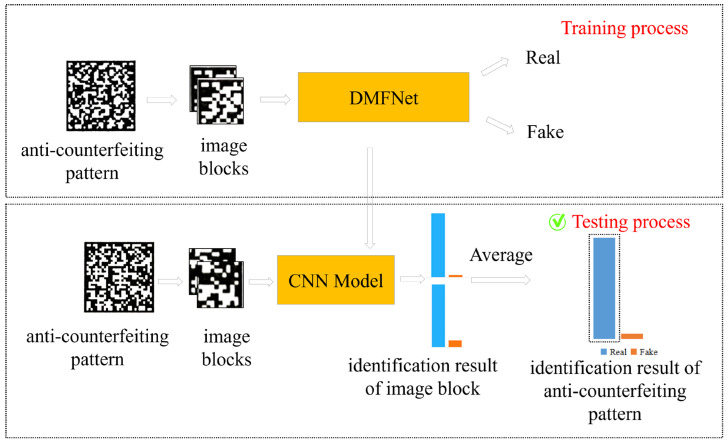
Flow chart of authenticity identification.

Figure 4 shows the overall flow of the proposed method, which can be divided into two stages of training and testing. First, the ACPs are extracted from the anti-counterfeiting QR codes to make a dataset, which mainly includes a training set, validation set and test set. Then, all ACPs in the dataset are divided into image blocks, DMFNet is trained using the training set and verification set, and the CNN model is obtained. Finally, the ACPs to be tested in the test set are divided into blocks and sent to the CNN model for prediction. The accuracy rates of all image blocks are obtained and then averaged, and thus the authenticity identification result is obtained.

The proposed authenticity identification scheme has the following characteristics:

(1) The ACP is divided into multiple image blocks by an overlapped blocking operation.
(2) The difference between the real ACP and the fake ACP is subtle, and the interference caused by the overall content of the ACP should be avoided during the authenticity identification process. Some researchers [36,43] suppressed the interference of image content in forensics by using special filters, such as Laplacian filters and high-pass filters, but the use of filters is a double-edged sword because they may also discard useful information. Since the convolutional layer can automatically learn the representation of useful features, this paper uses the convolutional self-learning method to amplify the traces left in the forgery process.
(3) A multi-feature fusion network with double-branch structure is proposed. The preprocessing layer in each branch uses convolution kernels of different sizes to extract multi-features representing subtle differences between a real ACP and a fake ACP. The structure of the two branches is the same, which is simple but effective. The dual-branch framework further enhances the feature extraction capability through a multi-feature fusion mechanism.

### 2.2. The Proposed Method

#### 2.2.1. Overlapping Blocks of Anti-Counterfeiting Patterns

In this paper, the self-designed ACP is used as research object, and its image size is much smaller than that of the whole anti-counterfeiting QR code. To ensure a sufficient amount of data, overlapped blocking techniques are used during processing.

**Figure 5:**
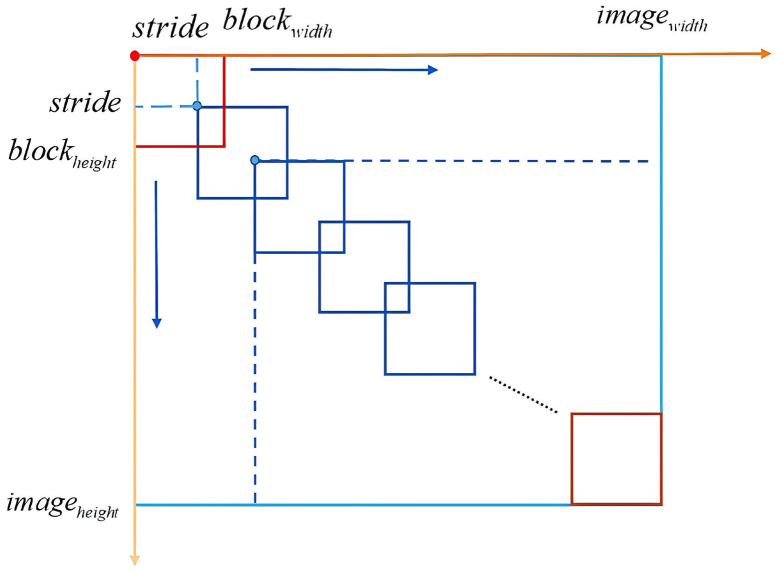
Schematic diagram of overlapping blocks.

Figure 5 shows the principle of the overlapped blocking technique, where stride is the step size, the size of the ACP is represented as $image_{height}\times image_{width}$, and the size of the image block is denoted as $block_{height}\times block_{weight}$, where height and width represent the height and width of the image, respectively. Since both the ACP and the image block are square, $image_{height}=image_{width},\;block_{height}=block_{width}$, and the number of image blocks is represented as $block_{row}\times block_{col}$, where row and col represents the number of rows and columns.

The corresponding formulas for calculating the stride value is shown in Formulas (1) and (2).

(1) $$Stride=\frac{image_{width}-block_{width}}{block_{row}-1}$$

(2) $$Stride=\frac{image_{height}-block_{height}}{block_{col}-1}$$

where N represents the number of blocks along the width side. Let $i\in \left\{1,\ldots ,block_{row}\right\},j\in \left\{1,\ldots ,block_{col}\right\}$; then, the coordinate $\left(x_{i,j},y_{i,j}\right)$ of the top left corner of the i-th row and j-th column is calculated as shown in Formulas (3) and (4):

(3) $$x_{i,j}=(j-1)\times Stride$$

(4) $$y_{i,j}=(i-1)\times Stride$$

The pseudocode of the overlapped blocking algorithm is given as follows (Algorithm 1):
**Algorithm 1** The pseudocode of the overlapped blocking algorithm**Input:** $image_{width}\times image_{height}$, $block_{width}\times block_{height}$, $block_{row}\times block_{col}$**Steps:**1: $For\;i=1\;to\;block_{row},\;do$ 2: $For\;j=1{\;to\;block}_{col},\;do$ 3: The step size stride is calculated from the image size of the ACP, the image size of block and the number of image blocks. 4:  The position coordinate of the upper left point of each image block is determined according the stride. 5: Save each image block. 6:    end 7:  end **Output:** Collection of all image blocks B, the number of image blocks row×col

#### 2.2.2. The Proposed DMFNet

**Figure 6:**
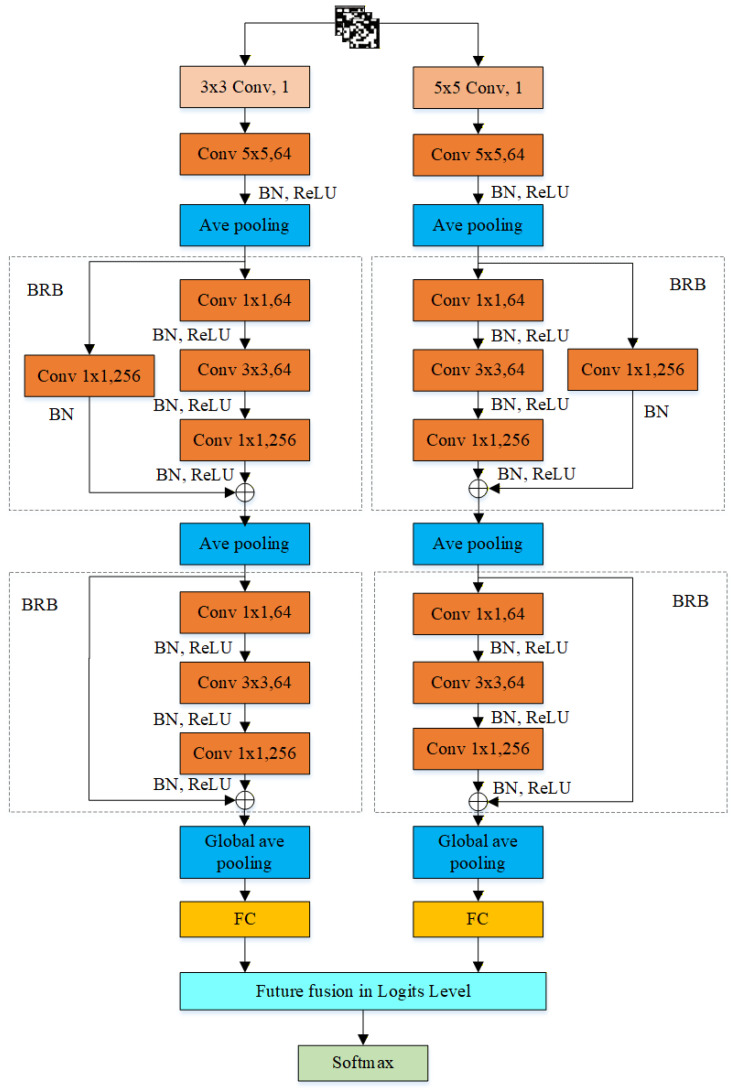
Structure of the DMFNet.

The structure of DMFNet is shown in Figure 6, where it can be seen that the DMFNet uses two branches for feature extraction. Each branch includes convolutional layers, batch normalization layers, ReLU activation layers, average pooling layers and bottleneck residual layers, and the structure of the two branches is the same regardless of the specific parameter settings of preprocessing layer.

The operation definition formula of the convolutional kernel is shown in Formula (5):

(5) $$F_{i+1}=\delta (k_{i+1}*F_{i}+B_{i+1})$$

where ∗ represents the convolution operator, $F_{i}$ represents the input feature map, $k_{i+1}$ represents the convolution kernel, $B_{i+1}$ represents the bias term, and $F_{i+1}$ represents the output feature map.

The feature map size for DMFNet is calculated as shown in Formula (6):

(6) $$F_{os}=\frac{F_{is}+2p-k}{s}+1$$

where $F_{is}$ represents the size of the input feature map, $F_{os}$ represents the size of the output feature map, *p* represents the padding size, *k* represents the size of the convolution kernel, and s represents the stride. The calculated value of $F_{os}$ is rounded down.

The parameters of the DMFNet are shown in Table 1. Both branches input single-channel grayscale images. From the calculation of Formula (3), it can be concluded that although the convolutional kernel sizes of the preprocessing convolutional layers in the two branches are different, after setting different padding sizes, their output sizes are the same, and this operation allows us to extract multiple features that indicate the difference between genuine and counterfeit, and also means the subsequent feature maps can be fused effectively.

The left branch of DMFNet is used as an example to calculate the change in the size of the feature map: The preprocessed image first goes through the first 5×5 convolution layer and pooling layer, and the size of the output feature map becomes 31×31×64. Then, the first bottleneck residual block is applied, which mainly consists of a 1×1 convolutional layer, batch normalization (BN) layer, ReLU layer, 3×3 convolution layer, BN layer, ReLU layer, 1×1 convolution layer, BN layer, ReLU layer and mean pooling layer. The number of feature maps output by the three convolutional layers is 64, 64, and 256; the kernel size of the average pooling layer is 5×5; the stride is 2; the pad is 0; and the size of the output feature map after pooling is 14×14. After that, the second bottleneck residual block is applied to further reduce the parameters. The global average pooling layer is used after the second bottleneck residual block, where the kernel size is 6×6, the stride is 1, and the pad is 0. The outputs of the two branches after global average pooling are separately transformed into one-dimensional representations and mapped to their respective classification outputs. Then, a feature fusion strategy is employed at the logits level to fuse the outputs of the two branches and obtain the final decision score. In this way, the complementary discriminative information extracted at different scales can be jointly utilized for authenticity identification. Finally, the category probabilities are calculated using the Softmax function, as shown in Formulas (7) and (8).

(7) $$p_{i}=\frac{e^{y_{i}}}{\sum_{j=1}^{n}e^{y_{j}}}$$

(8) $$y_{i}=y_{i}^{\left(1\right)}+y_{i}^{\left(2\right)}$$

where $p_{i}$ denotes the predicted probability of class i, and n denotes the number of categories. Since authenticity identification is a binary classification task, the value of n is set to 2. $y_{i}$ represents the fused logit of class i, while $y_{i}^{\left(1\right)}$ and $y_{i}^{\left(2\right)}$ denote the branch-wise logits produced by Branch 1 and Branch 2, respectively. The final decision score is obtained by fusing the logits from the two branches, and the category probabilities are then calculated using the SoftMax function. The parameters involved in the two branch-wise classification mappings are learned by Mini-Batch Gradient Descent [44].

## 3. Experimental Results and Analysis

### 3.1. Experiment Platform

The experiment platform, software and hardware settings are shown in Table 2. The deep learning framework used is Caffe; CUDA 9.0 and CUDNN 7.0.5 are used for GPU acceleration of CNNs.

### 3.2. Dataset Production

The specific production process of real and fake anti-counterfeiting QR codes is as follows:

1. Real anti-counterfeiting QR codes: The official authorized printer Toshiba e-studio 2051c-11606695 (Toshiba Tec Corporation, Tokyo, Japan) was used to print 48 anti-counterfeiting QR code images, 5 mobile phones were used to collect the real anti-counterfeiting QR code dataset, and a total of 240 images were obtained. The brands and models of the printer and smartphones are presented in Table 3 and Table 4, respectively.
2. Fake anti-counterfeiting QR codes: Eight sets of all-in-one printing and copying machines were used to forge by scanning and then printing. A total of 1920 fake anti-counterfeiting QR codes were obtained, and then 240 fake anti-counterfeiting QR codes were randomly selected.

The image size of all anti-counterfeiting QR codes was unified to 512 × 512, and then the ACPs were extracted. Since the position of the ACP in the anti-counterfeiting QR code is fixed, the ACP can be extracted by its length, width and the coordinates of the upper-left vertex. Table 5 shows the quantity distribution of the ACPs. Each ACP is divided into blocks by overlapping blocks, and the parameter setting are shown in Table 6.

The image size of the ACP is unified to 162×162, the stride is set to 14×14, an anti-counterfeiting pattern can be divided into 64 blocks, and the size of each image blocks is 64×64. The 480 original ACP images were first split at the image level into training, validation, and test subsets in a ratio of 3:1:1. Then, overlapping block extraction was conducted independently within each subset. As a result, a total of 30,720 image blocks were obtained, and the corresponding numbers are summarized in Table 7.

Table 8 shows examples of image blocks of real and fake ACPs. For the image block of the real ACP, its lines are relatively regular, while the fake ACP has uneven distribution of line thickness and prominent distortion due to the secondary random adsorption of the toner.

### 3.3. Performance Test of the Proposed Algorithm

After referring to relevant literature, we selected the commonly used Gray-Level Co-occurrence Matrix (GLCM) [45], Local Binary Pattern (LBP) [24], and Histogram of Oriented Gradient (HOG) [46] for comparison. These handcrafted feature extraction methods were used in conjunction with a Support Vector Machine (SVM) classifier, and a linear kernel was adopted. In addition, we selected several deep learning methods for comparison. Among them, Ashlesh [47] proposed a convolutional neural network for product authenticity identification, which can effectively distinguish subtle texture differences between genuine and counterfeit products. It was modified from AlexNet [48] by reducing the size of the convolution kernel and the stride of the first convolutional layer, thereby improving the extraction ability of fine-grained features. ResNet18 is widely used in the field of QR code identification [41,49,50], and was therefore also included for comparison. Meanwhile, ConvNeXt-Tiny [51], ViT-B/16 [52], and Swin-T [53], as representative stronger and more recent architectures, were also adopted as comparison methods. ConvNeXt-Tiny is a representative convolution-based backbone, while ViT-B/16 and Swin-T are two widely used Transformer-based visual models. All the above methods were tested on the ACP dataset, and are abbreviated as LBP_anti-pat_, GLCM_anti-pat_, HOG_anti-pat_, Ashlesh_anti-pat_, ResNet18anti-pat, ConvNeXt_anti-pat_, ViT-B_anti-pat_, Swin-T_anti-pat_, and DMFNet_anti-pat_, respectively. Table 9 shows the hyper-parameter settings of the proposed DMFNet.

In order to verify the advantages of the ACP designed in this paper in authenticity identification, we use the entire anti-counterfeiting QR code images to make a dataset for experimental comparison. The 480 anti-counterfeiting QR code images (240 genuine and 240 counterfeit) were first unified to 512 × 512 and then split at the original-image level into the training, validation, and test sets in a ratio of 3:1:1. After that, each QR code image in each subset was divided into 64 image blocks with a size of 64 × 64 using a non-overlapping block method. Experiments were also conducted using GLCM, LBP, HOG, Ashlesh, ResNet18, ConvNeXt, ViT-B, Swin-T, and the proposed DMFNet, which are abbreviated as GLCM_whole_, LBP_whole_, HOG_whole_, Ashlesh_whole_, ResNet18_whole_, ConvNeXt_whole_, ViT-B_whole_, Swin-T_whole_, DMFNet_whole_, respectively.

The confusion matrix [54] is used to evaluate the compared methods, as shown in Table 10. The true labels are represented by rows, and the predicted labels are represented by columns. The confusion matrix can be obtained by counting the true positive (TP), false positive (FP), false negative (FN) and true negative (TN).

**Figure 7:**
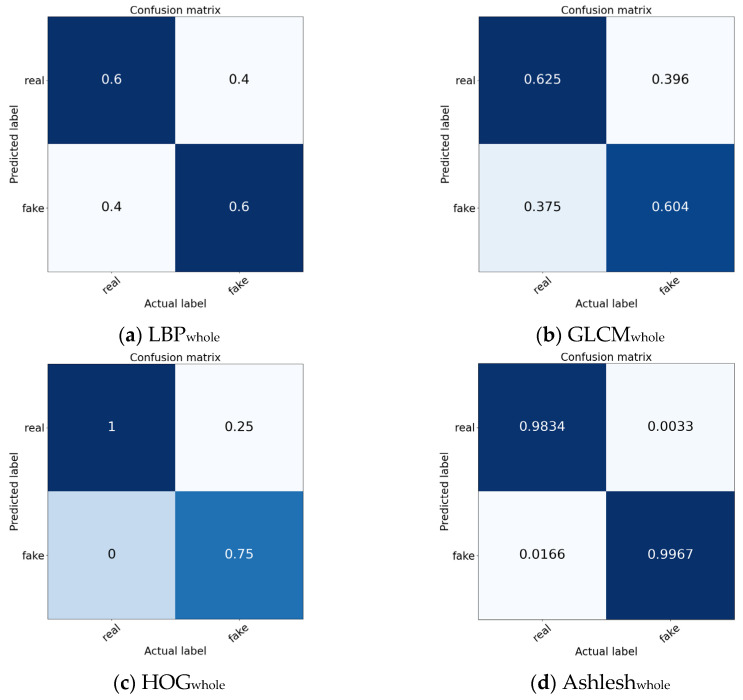

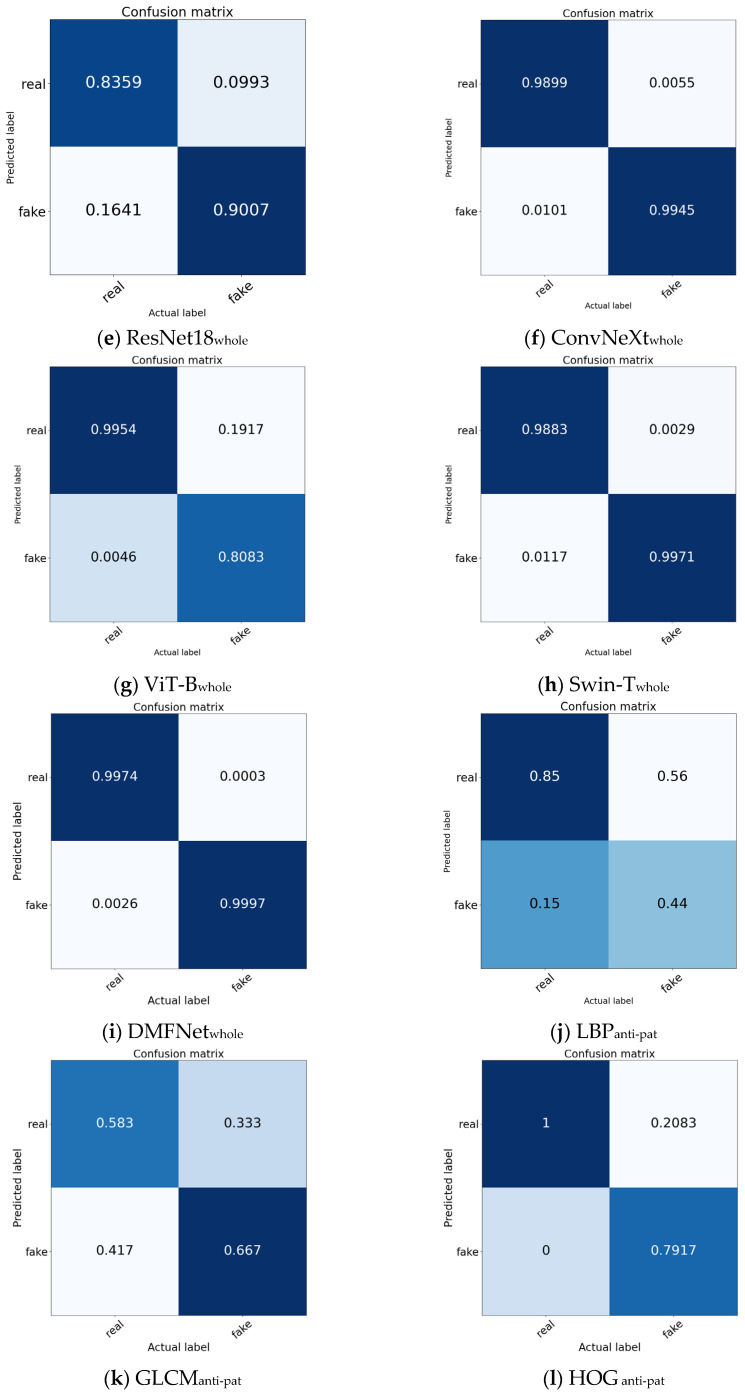

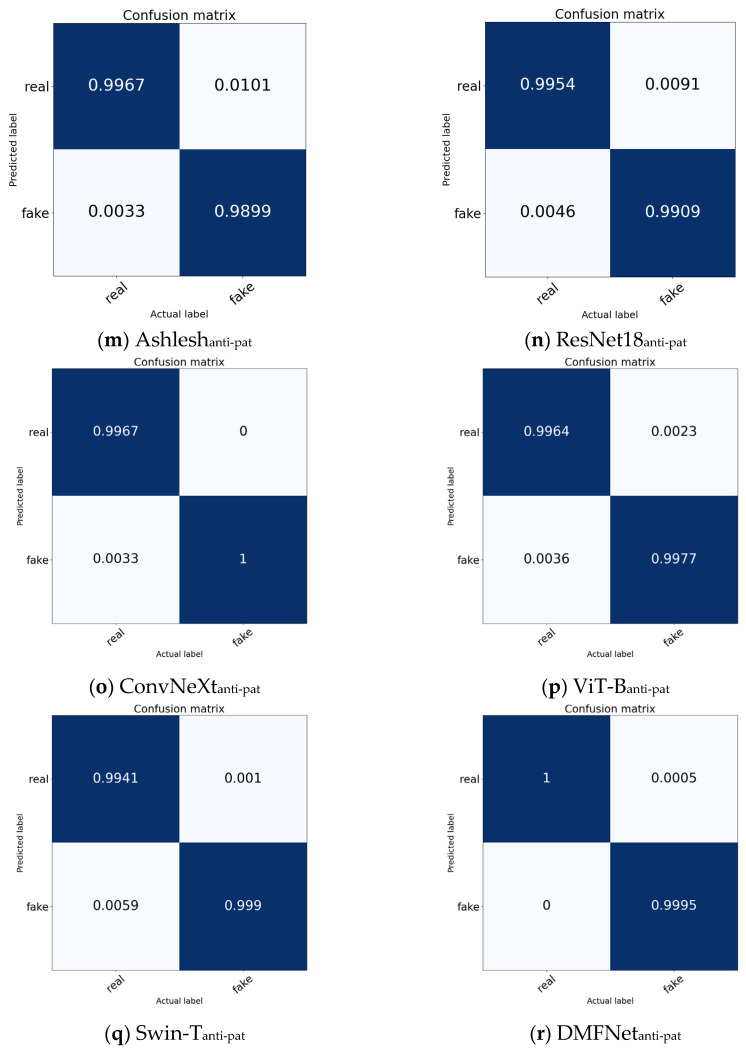
Confusion matrices of the compared methods on the entire anti-counterfeiting QR code dataset and the ACP dataset.

The confusion matrices of the compared methods on the entire anti-counterfeiting QR code dataset and the ACP dataset are shown in Figure 7.

Comparing the experimental results on the anti-counterfeiting QR code dataset and the ACP dataset, Figure 7 shows that the compared methods (GLCM, LBP, HOG, Ashlesh, ResNet18, ConvNeXt, ViT-B, Swin-T and DMFNet) perform better on the ACP dataset. This indicates that the fine texture of the ACP itself can highlight traces in the counterfeiting process, making it easier to accurately identify counterfeits. The proposed DMFNet achieves 100% identification accuracy in identifying real ACP categories and 99.95% accuracy in identifying fake ACP categories, demonstrating that it possesses the best feature extraction and authenticity identification capabilities.

**Figure 8:**
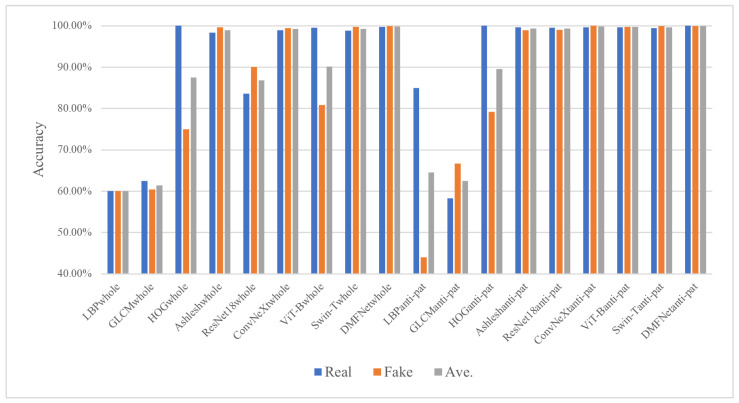
Comparison of the accuracy results of the compared methods on the entire anti-counterfeiting QR code dataset and the ACP dataset.

Then, the performance of DMFNet was tested using the accuracy of the real class, the accuracy of the fake class, and the average accuracy, as shown in Figure 8 and Table 11. The accuracy of the real class corresponds to the TP in the confusion matrix, the accuracy of the fake class corresponds to the TN in the confusion matrix, and the average accuracy is obtained by averaging the accuracy of the TP and the accuracy of the TN. The average accuracy is expressed as a combination of the mean (Ave.) and the standard deviation (Std. Dev.) [55].

Table 11 and Figure 8 compare handcrafted feature-based methods and deep learning models on the entire anti-counterfeiting QR-code dataset and the ACP dataset. The results clearly indicate that deep learning methods are more suitable for QR-code forgery detection than traditional LBP, GLCM, and HOG features combined with SVM. This is mainly because illegal copying introduces subtle visual degradations, such as edge blurring, ink diffusion, printing artifacts, and micro-texture distortion, which are difficult to characterize using handcrafted descriptors.

On the entire QR code dataset, traditional methods show limited performance, with LBPwhole and GLCMwhole achieving only 0.6000 and 0.6145 average accuracy, respectively. Although HOGwhole improves the accuracy to 0.8750, its relatively large standard deviation indicates unstable classification. In contrast, deep learning models achieve much higher performance. Among them, DMFNetwhole obtains the best result, with 0.9974 accuracy for genuine samples, 0.9997 accuracy for counterfeit samples, and an average accuracy of 0.9985 ± 0.0016. This demonstrates that DMFNet can effectively extract discriminative forgery-related features from the whole QR code image while maintaining high stability.

More importantly, the results on the ACP dataset further confirm the effectiveness of focusing on the anti-counterfeiting pattern region. Compared with the entire QR code image, the ACP region contains more concentrated and discriminative authentication information while reducing interference from irrelevant encoding modules and background structures. Traditional methods still perform poorly on this dataset, especially LBPanti-pat and GLCManti-pat, whose average accuracies are only 0.6450 and 0.6250, respectively. HOGanti-pat performs better but remains less stable due to its dependence on gradient-level information.

By contrast, deep learning models achieve consistently high performance on the ACP dataset. Ashleshanti-pat, ResNet18anti-pat, ConvNeXtanti-pat, ViT-Banti-pat, and Swin-Tanti-pat all obtain average accuracies above 0.99, showing that deep networks can effectively learn fine-grained differences between genuine and counterfeit anti-counterfeiting patterns. These results suggest that the ACP region provides stronger forensic cues for detecting copied QR codes than the whole QR code image.

The superior performance of DMFNet can be attributed to its multi-feature fusion mechanism. Unlike conventional single-backbone models, DMFNet integrates complementary features from different receptive fields and representation levels, enabling it to capture both local texture degradation and structural distortion caused by illegal copying. This design improves the model’s sensitivity to subtle counterfeit traces while preserving robust recognition of genuine samples. The very low standard deviation also indicates that DMFNet achieves balanced classification performance and does not show obvious bias toward either genuine or counterfeit samples.

In summary, the experimental results demonstrate that handcrafted feature-based methods are inadequate for robust QR code anti-counterfeiting verification, whereas deep learning methods provide significant improvements. Among all compared approaches, DMFNet achieves the best overall performance on the entire QR code dataset, particularly in counterfeit recognition accuracy and stability. These findings verify the effectiveness, robustness, and practical potential of DMFNet for fine-grained QR code forgery detection.

In addition to the above indicators, Precision, Recall and F1-score [56,57] are also effective indicators to measure the performance of classification algorithms. The F1-score is calculated based on precision and recall, as shown in Formula (9). Precision represents the proportion of correctly predicted real class samples out of all samples predicted as true class. Recall represents the proportion of real samples out of all predicted real samples. The F1-score is a harmonic average of Precision and Recall.

(9) $$F_{1}=2\times \frac{precision\times recall}{precision+recall}$$

The higher value of the three indicators, the better the performance of the authenticity identification algorithm.

**Figure 9:**
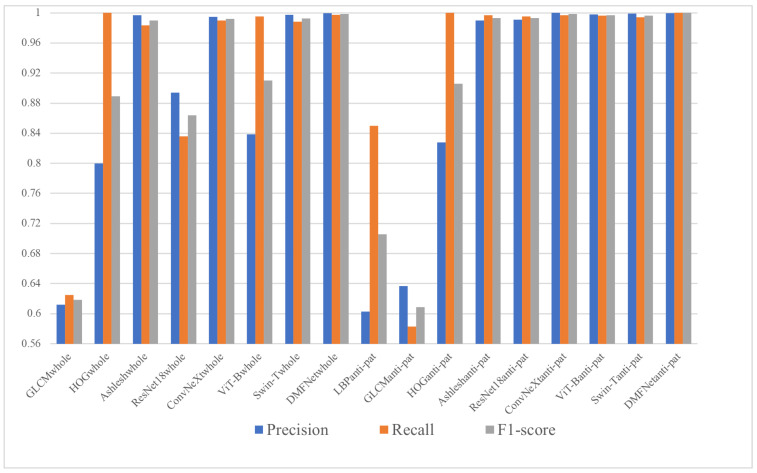
Comparison of Precision, Recall and F1-score of several methods.

Table 12 and Figure 9 present the Precision, Recall and F1-score of several methods on the whole QR code dataset and the anti-counterfeiting pattern dataset. The compared methods include traditional handcrafted feature descriptors combined with SVM, namely LBP, GLCM and HOG, as well as deep learning models, including Ashlesh, ResNet18, ConvNeXt, ViT-B, Swin-T and the proposed DMFNet.

Overall, deep learning methods achieve much better performance than traditional machine learning methods. On the whole QR code dataset, LBP and GLCM obtain relatively low F1-scores of 0.6 and 0.6185, respectively, indicating that simple texture statistics are insufficient to describe the subtle distortions caused by illegal copying. HOG performs better, with an F1-score of 0.8889 and a Recall of 1.0, suggesting that gradient information is useful for detecting certain structural differences. However, its Precision is only 0.8000, showing that handcrafted gradient features are still prone to false positives and lack strong discriminative capability.

Compared with handcrafted features, deep learning models show stronger representation ability. On the whole QR code dataset, Ashlesh, ConvNeXt, Swin-T and DMFNet all achieve F1-scores higher than 0.9900. Nevertheless, the performance of different deep models is not identical. ResNet18 obtains an F1-score of 0.8639, which indicates that a conventional lightweight CNN may not be sufficiently sensitive to fine-grained forgery traces. ViT-B achieves high Recall but relatively lower Precision, implying that global self-attention alone may be less stable when distinguishing weak local counterfeit artifacts. In contrast, DMFNet achieves the best overall performance, with Precision, Recall and F1-score values of 0.9997, 0.9974 and 0.9985, respectively. These results demonstrate that DMFNet can effectively capture discriminative features from whole QR code images while maintaining a good balance between false positives and false negatives.

The results on the anti-counterfeiting pattern dataset further demonstrate the importance of focusing on discriminative local regions. Most methods obtain better performance on the anti-counterfeiting pattern dataset than on the whole QR code dataset. For example, the F1-score of ResNet18 increases from 0.8639 to 0.9931, while that of ViT-B increases from 0.9102 to 0.9970. This improvement indicates that the anti-counterfeiting pattern region contains more concentrated and task-relevant forgery information. Compared with the whole QR code image, which includes many regular black-and-white modules and redundant background structures, the anti-counterfeiting pattern region better preserves micro-texture variations, edge degradation and printing artifacts caused by the scan–print–recapture process.

Among all methods evaluated on the anti-counterfeiting pattern dataset, DMFNet again achieves the best result, with a Precision of 0.9995, a Recall of 1.0000 and an F1-score of 0.9998. This performance is higher than that of ConvNeXt, ViT-B and Swin-T, whose F1-scores are 0.9983, 0.9970 and 0.9965, respectively. In particular, the perfect Recall of DMFNet indicates that it can successfully identify all target samples in this evaluation, while its extremely high Precision shows that this improvement is not achieved by increasing false positives. Such a balanced performance is highly important for practical QR code anti-counterfeiting applications, where both missed counterfeit samples and false rejection of genuine samples may lead to security risks or poor user experience.

In summary, the experimental results show that traditional handcrafted features have limited capability in modeling complex counterfeit traces, whereas deep learning methods can learn more powerful and discriminative representations. More importantly, DMFNet consistently achieves the highest F1-scores on both datasets, reaching 0.9985 on the whole QR code dataset and 0.9998 on the anti-counterfeiting pattern dataset. These results verify the superiority and robustness of DMFNet in QR code authenticity verification. The strong performance of DMFNet can be attributed to its effective feature fusion strategy, which enables the model to synergistically capture fine-grained forgery traces across different scales, thereby improving the reliability of genuine–counterfeit classification.

## 4. Discussion

### 4.1. Selection of Convolutional Kernels in Preprocessing Convolutional Layers

In the field of image forensics, special preprocessing convolutional layers can suppress semantic content and capture subtle forgery traces. For the dual-branch structure in this paper, we tried several selection methods for the convolution kernel size in the two branches: 3 × 3 and 5 × 5 convolution kernels, 3 × 3 and 7 × 7 convolution kernels, and 5 × 5 and 7 × 7 convolution kernels. The experimental results are shown in Table 13.

As shown in Table 13, experimenting with different convolutional kernels in the preprocessing layer yields subtle differences in results. When the two branches are set to 3 × 3 and 7 × 7, respectively, the overall accuracy is 99.89%; when set to 5 × 5 and 7 × 7, respectively, the overall accuracy is 99.63%; and when the two branches are set to 3 × 3 and 5 × 5, respectively, the accuracy reaches 99.98%. These results can be attributed to the inherent characteristics of anti-counterfeiting pattern authentication. Copying and printing artifacts, such as toner scattering, edge blurring, and halftone patterns, typically manifest as high-frequency micro-textures within a specific spatial range. The 3 × 3 and 5 × 5 convolutional kernels are well suited for capturing these local pixel-level discontinuities, while the larger 7 × 7 kernel tends to produce a smoothing effect, which acts like a low-pass filter, diluting subtle forensic features and introducing redundant structural information from the QR code module. Therefore, multi-scale fusion of 3 × 3 and 5 × 5 branches most effectively balances the extraction of discriminative high-frequency residual features with the suppression of macroscopic image content interference.

### 4.2. Resilience to Blurring

When capturing anti-counterfeiting patterns with a smartphone camera, blurring due to motion is unavoidable. To evaluate the adaptability of the proposed DMFNet under blurred conditions, we added different degrees of motion blur to the captured anti-counterfeiting patterns. Specifically, we set five different motion blur kernel lengths: 2, 4, 6, 8, and 10. The angle of the blur kernel was uniformly set to 45 degrees. The experimental results are shown in Table 14.

The results in Table 14 show a clear trade-off between image sharpness and authentication accuracy. When the blur length is 4, the model maintains a high accuracy of 96.09%; however, when the blur length exceeds 6, performance drops significantly, with accuracy decreasing to 55.08% at a blur length of 10, approaching random levels. This sharp decline highlights the proposed method’s dependence on high-frequency forensic residual features. Motion blur physically acts as a spatial low-pass filter, blurring the random toner distribution and edge irregularities that distinguish between genuine and fake QR codes. When the blur displacement exceeds the spatial scale of these micro-textures, the discriminative “printed fingerprint” is irreversibly lost, causing the feature representations of real and fake QR codes to become consistent.

### 4.3. Resilience to Illumination

To evaluate the robustness of the proposed DMFNet under different illumination conditions, we simulated the image acquisition process of a smartphone under varying illumination intensities. Specifically, we applied illumination transformations to the test images to simulate different exposure conditions during smartphone photography. We considered four illumination intensities: 0.6 times, 0.8 times, 1.2 times, and 1.4 times the original illumination intensity. Then, we used four sets of processed test images to evaluate the performance of the DMFNet. The experimental results are shown in Table 15.

The experimental results in Table 15 indicate that the proposed DMFNet is sensitive to changes in illumination intensity. Under slight underexposure, when the illumination coefficient is 0.6, the model still achieves an accuracy of 87.45%. When the coefficient is 0.8, the accuracy further increases to 97.28%, while the baseline condition of 1.0 gives the best performance of 99.98%. However, when the illumination becomes stronger than the baseline, the performance drops significantly. Specifically, the accuracy decreases to 75.41% at the coefficient of 1.2 and further falls to 50.28% at 1.4, approaching the level of random guessing. These results suggest that overexposure has a more severe effect on DMFNet than slight underexposure, probably because excessively strong illumination causes saturation or distortion of the fine discriminative textures in the anti-counterfeiting pattern.

### 4.4. Cross-Validation and Ablation Study

To further assess the robustness of the proposed model, a customized five-run cross-validation strategy was conducted at the original-image level. Specifically, because the number of genuine samples was limited, the genuine images were reused across runs, whereas the counterfeit images were divided as evenly as possible into five subsets. In each run, one counterfeit subset was used for evaluation, and the counterfeit images used for testing in that run were excluded from training. Image blocking was performed only after the image-level split, thereby avoiding leakage between training and test samples.

As shown in Table 16 and Table 17, the full model consistently achieved accuracy of 99.98% across the five runs and also exhibited the smallest fluctuation, which corresponds to the results in Table 11 and Table 12. This result indicates that the proposed method not only provides high recognition accuracy but also maintains stable behavior under varying counterfeit-image partitions.

The comparison between the full model and the Feature Stacking variant further clarifies the role of the proposed dual-branch decision strategy. The feature stacking method simply concatenates the outputs of the two branches after global average pooling at the feature level and sends the combined representation to a single classifier for final prediction. Although direct stacking of the two branch features still produces strong results, it remains consistently inferior to the full model and shows weaker stability. This suggests that the gain is not merely due to combining two sets of features, but to a more effective interaction and decision process that better exploits the complementarity between the two branches.

A clearer degradation is observed when the preprocessing layer is removed or when the network is simplified to a single branch. The No Preprocess variant shows the most obvious performance drop and the largest fluctuation, indicating that the preprocessing layer is important for enhancing subtle authenticity-related traces before deep feature extraction. Similarly, both single-branch variants perform worse than the full model, and the 5 × 5 branch, although slightly better than the 3 × 3 branch, still cannot match the complete architecture. Taken together, these results indicate that the best performance is achieved only when the preprocessing layer, the dual-branch structure, and the proposed decision strategy are used jointly.

### 4.5. Cross-Domain Verification

To further evaluate the generalization ability of DMFNet beyond the conventional in-domain setting, cross-domain experiments were conducted based on the ACP datasets. Following the same principle used in the main experiments, the ACP datasets was first divided at the original image level into training, validation, and test subsets in a ratio of 3:1:1, and ACP patch generation was then performed within each subset to avoid information leakage. On this basis, two more challenging protocols, namely cross-device and cross-printer evaluation, were further constructed, as summarized in Table 18, Table 19, Table 20 and Table 21. In these tables, P0–P8 denote nine different printers, and the corresponding printer identities are detailed in Table 3. In essence, these experiments were designed to examine whether the proposed model can still maintain reliable authentication performance when either the image acquisition domain or the counterfeit printing domain is unseen during training.

In the cross-device setting, all printer domains were preserved, while the image acquisition domain was changed across groups. Specifically, one smartphone was left out as the test domain in each group alone, and the remaining devices were just used for training and validation. This protocol is more demanding than standard evaluation because the model must generalize to an unseen imaging device with different sensor characteristics, noise patterns, and imaging responses. As reported in Table 19, the proposed method still achieved strong overall performance, with an average accuracy of 96.13%. Although a certain degree of variation can be observed across groups, the overall results indicate that DMFNet remains effective under device shift and is able to capture features that are not overly dependent on a specific acquisition device.

In the cross-printer setting, the genuine samples generated by the official printer were retained in every group, whereas the counterfeit domains were reorganized across different training and test splits. More specifically, three counterfeit printers were reserved exclusively for testing in each group, while the remaining counterfeit printers were used for training and validation. This design better reflects practical anti-counterfeiting scenarios, in which the source of counterfeit printing is usually unknown in advance. As shown in Table 21, the proposed method achieved even more stable performance under this protocol, reaching an average accuracy of 99.26%. Compared with the cross-device task, the cross-printer results show smaller fluctuations across groups, suggesting that the ACP-related forgery traces learned by DMFNet are highly transferable across unseen counterfeit printing sources.

Overall, the cross-domain results demonstrate that DMFNet is not limited to the closed-set ACP classification setting, but also maintains strong performance when facing previously unseen devices or counterfeit printers. These findings confirm the robustness of the proposed method and further support its practical potential in real-world QR code anti-counterfeiting applications.

## 5. Conclusions

This study proposed a QR code authentication framework that integrates an anti-counterfeiting pattern with a Double-branch Multi-feature Fusion Network. The designed anti-counterfeiting pattern introduces fine-grained stochastic textures into conventional QR codes, making the micro-scale distortions caused by illegal scan–print copying more distinguishable. To further exploit these subtle forensic traces, overlapping block processing was adopted to expand local texture samples, and DMFNet was developed to learn complementary multi-scale features through two preprocessing convolution branches.

The experimental results answer the central research question of this study: whether ACP-based local texture cues and multi-feature fusion can improve QR code authenticity verification. Compared with handcrafted descriptors and representative deep learning models, DMFNet achieved the best overall performance on both the whole QR code dataset and the ACP dataset, with an average accuracy of 99.98% and an F1-score of 0.9998 on the ACP dataset. The ablation study further confirmed that the preprocessing layer, dual-branch structure, and feature fusion strategy jointly contribute to the performance improvement. In addition, the cross-domain experiments demonstrated promising generalization ability, achieving average accuracies of 96.13% in cross-device evaluation and 99.26% in cross-printer evaluation.

These findings indicate that the proposed ACP and DMFNet framework can effectively capture copying-induced texture degradation and has practical potential for low-cost, smartphone-based QR code anti-counterfeiting applications. Nevertheless, the robustness experiments also show that severe motion blur and strong overexposure may weaken the discriminative texture information and reduce recognition accuracy. Therefore, future work should focus on improving robustness under complex acquisition conditions, such as blur, illumination variation, compression, geometric misalignment, and cross-session scenarios. In addition, larger-scale datasets covering more printers, mobile devices, materials, and real-world usage environments should be constructed to further validate and enhance the practical reliability of the proposed method.

## Figures and Tables

**Table 1:** Parameter configuration of DMFNet.

Module	Output Feature Map Size	Branch 1	Branch 2
Input	64 × 64 × 1		
Pre_conv	64 × 64	3 × 3 × 1 Stride: 1 Pad: 1	5 × 5 × 1 Stride: 1 Pad: 2
Conv_1	64 × 64	data	data
Ave pooling	31 × 31	5 × 5	5 × 5
		Stride: 2 Pad: 0	Stride: 2 Pad: 0
Bottleneck1_	31 × 31	1 × 1 × 64 Stride:1 3 × 3 × 64 Stride:1 1 × 1 × 256 Stride:1	1 × 1 × 64 Stride:1 3 × 3 × 64 Stride:1 1 × 1 × 256 Stride:1
Ave pooling	14 × 14	5 × 5 Stride: 2 Pad: 0	5 × 5 Stride: 2 Pad: 0
Bottleneck2_	14 × 14	1 × 1 × 64 Stride:1 3 × 3 × 64 Stride:1 1 × 1 × 256 Stride:1	1 × 1 × 64 Stride:1 3 × 3 × 64 Stride:1 1 × 1 × 256 Stride:1
Global ave pooling	9 × 9	6 × 6 Stride: 1 Pad: 0	6 × 6 Stride: 1 Pad: 0
Softmax	1×n		

**Table 2:** Experiment platform.

Notebook model:	HP OMEN 17-w119TX
CPU	Intel Core i7-7700HQ (2.80 GHz, 16 GB),
GPU	Nvidia Geforce GTX1070 (8 GB)
Operating system	Windows10
Deep Learning framework	Caffe
CUDA version	9.0
CUDNN version	7.0.5

**Table 3:** The brand and model of printers.

Numbering	Brand	Model	Quantity
0	Canon	Image Runner ADVANCE 6575 (Canon Inc., Tokyo, Japan)	48
1	Epson	L15168 (Seiko Epson Corporation, Suwa, Japan)	48
2	Lenovo	MD7600 (Lenovo Group Limited, Beijing, China)	48
3	RICOH	Aficio MP7502-I (Ricoh Company, Ltd., Tokyo, Japan)	48
4	RICOH	Aficio MP7502-II (Ricoh Company, Ltd., Tokyo, Japan)	48
5	RICOH	Aficio MP8001 (Ricoh Company, Ltd., Tokyo, Japan)	48
6	RICOH	Aficio MP7001 (Ricoh Company, Ltd., Tokyo, Japan)	48
7	RICOH	Imagio MP 7502 (Ricoh Company, Ltd., Tokyo, Japan)	48
8 (Official)	Toshiba	e-studio 2051c-11606695 (Toshiba Tec Corporation, Tokyo, Japan)	48
Total			432

**Table 4:** The brand and model of smartphones.

Numbering	Brand	Model	Quantity
1	Huawei	Mate40 Pro	432
2	Huawei	Nova5 Pro	432
3	iPhone	X	432
4	iPhone	13	432
5	Redmi	K30	432
Total			2160

**Table 5:** Quantity distribution of ACPs.

Category	Quantity
Real	240
Fake	240
Total	480

**Table 6:** Parameters of overlapping blocks.

Parameters	Settings
$Image_{width}\times Image_{height}$	162×162
stride	14×14
$block_{row}\times block_{col}$	8×8
$block_{width}\times block_{height}$	64×64

**Table 7:** Number distribution after the anti-counterfeiting pattern block dataset.

Dataset	Real	Fake	Total
Training set	9216	9216	18,432
Verification set	3072	3072	6144
Test set	3072	3072	6144
Total	15,360	15,360	30,720

**Table 8:** Image block examples of real and fake anti-counterfeiting patterns.

Classes	Samples
Real	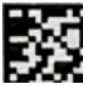 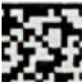
Fake	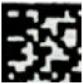 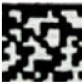

**Table 9:** The hyper-parameter settings of the proposed DMFNet.

Hyper-Parameter	Settings
Solver type	Stochastic gradient descent
Base learning rate	0.01
Policy	Step down
Step Size	33%
Gamma	0.1
Epochs	50
Iterations	12,000

**Table 10:** Confusion matrix.

	Actual	Real	Fake
Predicted			
Real		TP	FP
Fake		FN	TN

**Table 11:** Comparison of the accuracy results of the compared methods on the entire anti-counterfeiting QR code dataset and the ACP dataset.

Dataset Type	Methods	Accuracy		
		Real	Fake	Ave. ± Std. Dev.
The entire anti-counterfeiting QR code dataset	LBP_whole_	0.6	0.6	0.6 ± 0
	GLCM_whole_	0.625	0.604	0.6145 ± 0.0148
	HOG_whole_	1	0.75	0.875 ± 0.1768
	Ashlesh_whole_	0.9834	0.9967	0.9901 ± 0.0094
	ResNet18_whole_	0.8359	0.9007	0.8683 ± 0.0458
	ConvNeXt_whole_	0.9899	0.9945	0.9922 ± 0.0033
	ViT-B_whole_	0.9954	0.8083	0.9019 ± 0.1323
	Swin-T_whole_	0.9883	0.9971	0.9927 ± 0.0062
	DMFNet_whole_	0.9974	0.9997	0.9985 ± 0.0016
ACP dataset	LBP_anti-pat_	0.85	0.44	0.645 ± 0.290
	GLCM_anti-pat_	0.583	0.667	0.625 ± 0.0594
	HOG_anti-pat_	1	0.7917	0.8958 ± 0.1473
	Ashlesh_anti-pat_	0.9967	0.9899	0.9933 ± 0.0048
	ResNet18_anti-pat_	0.9954	0.9909	0.9932 ± 0.0032
	ConvNeXt_anti-pat_	0.9967	1	0.9984 ± 0.0023
	ViT-B_anti-pat_	0.9964	0.9977	0.9971 ± 0.0009
	Swin-T_anti-pat_	0.9941	0.9990	0.9966 ± 0.0035
	DMFNet_anti-pat_	1	0.9995	0.9998 ± 0.0004

**Table 12:** Comparison of Precision, Recall and F1-score of several methods.

Methods	Precision	Recall	F1-Score
LBP_whole_	0.6	0.6	0.6
GLCM_whole_	0.6121	0.625	0.6185
HOG_whole_	0.8	1	0.8889
Ashlesh_whole_	0.9967	0.9834	0.99
ResNet18_whole_	0.8938	0.8359	0.8639
ConvNeXt_whole_	0.9945	0.9899	0.9922
ViT-B_whole_	0.8385	0.9954	0.9102
Swin-T_whole_	0.9971	0.9883	0.9927
DMFNet_whole_	0.9997	0.9974	0.9985
LBP_anti-pat_	0.6028	0.85	0.7054
GLCM_anti-pat_	0.6365	0.583	0.6086
HOG_anti-pat_	0.8276	1	0.9057
Ashlesh_anti-pat_	0.99	0.9967	0.9933
ResNet18_anti-pat_	0.9909	0.9954	0.9931
ConvNeXt_anti-pat_	1	0.9967	0.9983
ViT-B_anti-pat_	0.9977	0.9964	0.9970
Swin-T_anti-pat_	0.9990	0.9941	0.9965
DMFNet_anti-pat_	0.9995	1	0.9998

**Table 13:** Selection of convolutional kernels in preprocessing convolutional layers.

Branch 1	Branch 2	Accuracy (%)
3 × 3	7 × 7	99.89
5 × 5	7 × 7	99.63
3 × 3	5 × 5	99.98

**Table 14:** Performance of DMFNet under different degrees of motion blur.

Motion Blur Kernel Size	Accuracy (%)
2 × 2	99.98
4 × 4	96.06
6 × 6	69.53
8 × 8	58.98
10 × 10	55.08

**Table 15:** Performance of DMFNet under different degrees of motion blur.

Illumination Coefficient	Accuracy (%)
0.6	87.45
0.8	97.28
1.0 (Baseline)	99.98
1.2	75.41
1.4	50.28

**Table 16:** Structural settings of different ablation variants.

Model	Preprocessing Layer	Branch Setting	Branch Combination
Full model	Yes	Dual branch (3 × 3 + 5 × 5)	Feature fusion
Feature Stacking	Yes	Dual branch (3 × 3 + 5 × 5)	Feature stacking
No Preprocess	No	Dual branch (3 × 3 + 5 × 5)	Feature fusion
Single-branch 3 × 3	Yes	Single branch (3 × 3)	None
Single-branch 5 × 5	Yes	Single branch (5 × 5)	None

**Table 17:** Quantitative results of the ablation study.

Model	Overall Acc (%)	CV (%)
Full model (proposed)	99.98 ± 0.0004	0
Feature Stacking	99.91 ± 0.0706	0.07
No Preprocess	99.22 ± 0.5094	0.51
Single-branch 3 × 3	99.91 ± 0.0811	0.08
Single-branch 5 × 5	99.95 ± 0.0557	0.06

**Table 18:** Cross-device task assignment.

Group	Training Devices	Test Device
Group1	Huawei Nova5 (Huawei Device Co., Ltd., Shenzhen, China), iPhone X (Apple Inc., Cupertino, CA, USA), iPhone 13 (Apple Inc., Cupertino, CA, USA), Redmi K30 5G (Xiaomi Corporation, Beijing, China)	Huawei Nova6 (Huawei Device Co., Ltd., Shenzhen, China)
Group2	Huawei Nova6, iPhone X, iPhone 13, Redmi K30 5G	Huawei Nova5
Group3	Huawei Nova6, Huawei Nova5, iPhone 13, Redmi K30 5G	iPhone X
Group4	Huawei Nova6, Huawei Nova5, iPhone X, Redmi K30 5G	iPhone 13
Group5	Huawei Nova6, Huawei Nova5, iPhone X, iPhone 13	Redmi K30 5G

**Table 19:** Cross-device evaluation results.

Group	Overall Acc. (%)	True Acc. (%)	Fake Acc. (%)	F1-Score
Group1	99.95	99.97	99.93	0.9995
Group2	96.76	93.52	100.00	0.9686
Group3	97.90	95.80	100.00	0.9794
Group4	99.20	99.51	98.89	0.9920
Group5	86.85	73.70	100.00	0.8838
Mean ± Std. Dev.	96.13 ± 5.33	92.50 ± 10.84	99.76 ± 0.49	0.9647 ± 0.0467

**Table 20:** Cross-printer task assignment.

Group	Training Printers	Test Printers
Group1	P3, P4, P5, P6, P7, P8	P0, P1, P2, P8
Group2	P0, P1, P2, P6, P7, P8	P3, P4, P5, P8
Group3	P1, P2, P3, P4, P5, P8	P0, P6, P7, P8
Group4	P0, P2, P4, P5, P7, P8	P1, P3, P6, P8
Group5	P0, P1, P3, P5, P6, P8	P2, P4, P7, P8
Group6	P1, P2, P3, P4, P6, P8	P0, P5, P7, P8

**Table 21:** Cross-printer evaluation results.

Group	Overall Acc. (%)	True Acc. (%)	Fake Acc. (%)	F1-Score
Group1	99.13	99.87	98.39	0.9912
Group2	99.90	99.79	100.00	0.9990
Group3	100.00	100.00	100.00	1.0000
Group4	97.06	100.00	94.11	0.9697
Group5	99.93	99.87	100.00	0.9993
Group6	99.52	99.84	99.19	0.9952
Mean ± Std. Dev.	99.26 ± 1.13	99.90 ± 0.09	98.62 ± 2.30	0.9924 ± 0.0116

## Data Availability

The data supporting this study were generated using multiple commercial printers and mobile devices. Due to privacy and security restrictions, the dataset is not publicly available. Data may be obtained from the corresponding author upon reasonable request.
